# Regulated growth of diatom cells on self-assembled monolayers

**DOI:** 10.1186/1477-3155-5-2

**Published:** 2007-03-23

**Authors:** Kazuo Umemura, Tomoaki Yamada, Yuta Maeda, Koichi Kobayashi, Reiko Kuroda, Shigeki Mayama

**Affiliations:** 1 Kamoshita Planning, SP1112-5-15-1, Ginza, Chuo-ku, Tokyo 104-8238, Japan; 2 Musashi Institute of Technology, 1-28-1 Tamazutsumi, Setagaya, Tokyo 158-8557, Japan; 3 The University of Tokyo, 3-8-1 Komaba, Muguro-ku, Tokyo 153-8902, Japan; 4 Kuroda Chiromorphology Project, ERATO-SORST, 4-7-6 Park Building, Komaba, Meguro-ku, Tokyo 153-0041, Japan; 5 Tokyo Gakugei University, Koganei, Tokyo 184-8511, Japan

## Abstract

We succeeded in regulating the growth of diatom cells on chemically modified glass surfaces. Glass surfaces were functionalized with -CF_3_, -CH_3_, -COOH, and -NH_2 _groups using the technique of self-assembled monolayers (SAM), and diatom cells were subsequently cultured on these surfaces. When the samples were rinsed after the adhesion of the diatom cells on the modified surfaces, the diatoms formed two dimensional arrays; this was not possible without the rinsing treatment. Furthermore, we examined the number of cells that grew and their motility by time-lapse imaging in order to clarify the interaction between the cells and SAMs. We hope that our results will be a basis for developing biodevices using living photosynthetic diatom cells.

## Background

Diatoms are one of the most major microalgae that are found everywhere – in seas, lakes, and rivers [1-4]. It is known that 25% of the O_2 _production on earth and 40% of the carbon fixation in the ocean are carried out by the photosynthesis of diatoms [1-4]. Furthermore, the cell wall of diatoms is decorated with ornamentations of various shapes that range from rib-like structures to well-organized nanoporous holes [5-7]. Hence, diatom shells are commonly used for filters [8], carriers [9], supports for chromatography [10], and building materials [11].

Because the diatom and its cell wall are very popular and because it is important for its use in bioreactors and as nanoporous material, the structures and functions of diatom cells have been intensively studied. For example, structural studies of diatom shells by using scanning electron microscopy (SEM) or atomic force microscopy have been carried out by many researchers [12-17]. From the biological viewpoint, the sequencing of the entire diatom genome was one of the recent remarkable projects [18]. However, few studies have proposed a technique that involves combining diatoms with nanotechnology. A pioneer study by Lebeau *et al*. reported on the fabrication of a photosynthetic biodevice using living diatoms [19]. They revealed that diatom cells can be cultured on agar films that are prepared on a glass surface, and that these cells can perform photosynthesis. Although this was an important study on developing biodevices by using living diatoms, no microscopic characterization of the device was included in the paper. To date, no other study has reported the development of biodevices by using living diatoms.

As related works, several papers that analyzed the motility of diatom cells by using microscopes could be found [10-24]. For example, Cohn *et al*. described that environmental factors affect diatom motility [20]. And Holland *et al*. found that the strength of the adhesion of the diatoms onto a surface is independent of their motility [24]. Although diatom motility has been an attractive subject of research for pure scientists, the obtained knowledge has not been applied to the development of biodevices involving the use of diatoms.

On the other hand, self-assembled monolayers (SAMs) are one of the most useful techniques used in nanotechnology [25-27]. Organosilane molecules bind to Si surfaces via Si-O-Si bonding [26]. Thiol molecules bind to metal surfaces such as an evaporated Au surface via metal-S bonding [27]. As a result, the silane or thiol molecules form self-assembled monolayers on the substrate surfaces.

The SAM technique has been used for various applications in biology. Typically, a mica surface that are functionalized with 3-aminopropyltriethoxysilane (APS), known as AP-mica, is used as the surface on which biomolecules are attached [28-34]. For example, DNA molecules are firmly attached onto an AP-mica surface because the DNA and the amino group of APS have negative and positive charges, respectively, under neutral pH conditions. Using this mechanism, Lyubchenko *et al*. successfully prepared a stable DNA sample for atomic force microscopy (AFM), and observed individual DNA molecules [28-30]. We also reported the AFM imaging of DNA fragments by using AP-mica [31-34]. Furthermore, the AP-mica that was prepared with diluted APS solution helped regulating adhesion force between DNA molecules and the AP-mica surface [33]. SAMs prepared with dilute APS whose surfaces were not entirely covered were effective in controlling DNA adhesion to the mica surface.

Finlay *et al*. employed alkanethiolate SAMs on a gold surface to study the adhesion strength of diatom cells to SAMs [35]. Their results clearly demonstrated that the adhesion of diatom cells (*Amphora*) was affected by the wettability of SAMs. Their work involving the combination of SAM techniques and cell biology in pioneering although they focused on studying the adhesion strength between SAMs and diatoms and not on the growth of diatom cells on SAMs. In general, cell researches but in diatom researches, many examples of cell adhesion onto chemically modified surfaces have been reported [36-40].

In this paper, we demonstrated the control of cell growth on chemically functionalized glass surfaces. A glass surface was functionalized by several kinds of SAMs that were prepared with diluted silane compounds, and diatom cells were then cultured on the surfaces in order to realize densely-packed cell arrays on the surfaces.

## Results and discussion

**Figure 1:**
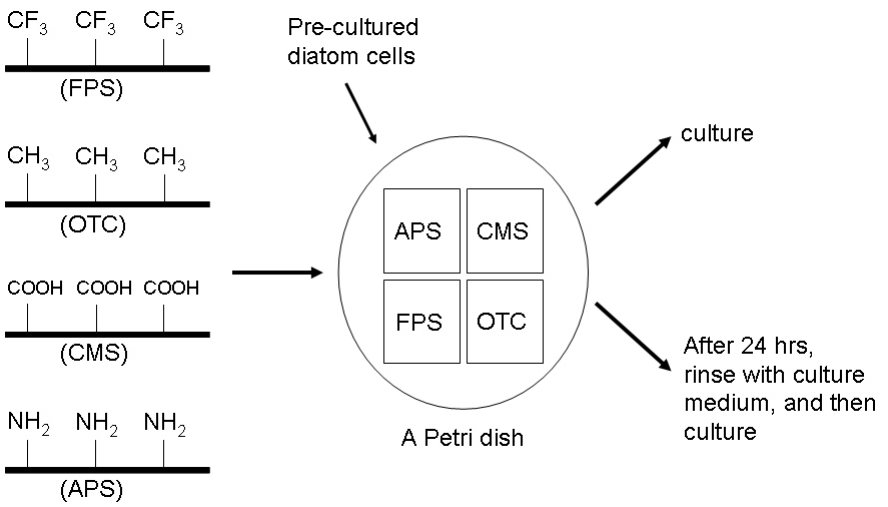
Schematic representation of the experiments. Glass surfaces were modified with self-assembled monolayers of FPS, APS, OTC, and CMS; these were then put into one petri dish. After one day of incubation, some of the samples were rinsed with the culture medium in order to remove any unattached diatom cells.

Figure 1 shows a schematic view of our experiments. Glass surfaces were functionalized with self-assembled monolayers by using organosilanes as described previously [26-34]. The glass surfaces were immersed in 1% silane solutions, and baked for 1 h at 90°C after rinsing. It is known that baking is an important procedure to complete chemical reaction at the surface and to remove the excess silane molecules [24]. Subsequently, the glass surfaces that were modified with 3,3,3-(trifluoropropyl)trimethoxysilane (FPS, -CF_3_), 7-Octenyltrichlorosilane (OTC, -CH_3_), 2-(carboxymethylthio)ethyl 3-trimethylsilane (CMS, -COOH), and 3-Aminopropyltriethoxysilane (APS, -NH_2_) were placed in a polystyrene dish. Since the four samples were placed in one dish, we could assume that the culture conditions of the four samples were identical.

After filling the dishes with fifty ml of the culture medium, a precultured diatom suspension was dropped into the dishes. The chemically modified glasses were completely submerged in the culture medium. Some of the dishes were cultured without any other treatment, and the others were rinsed with the culture medium after 24 h in order to remove the unattached diatom cells. For rinsing, samples were moved to another Petri dish that was filled with 50 ml of the culture medium. After keeping one minute without shaking, the samples moved again to another Petri dish that was filled with 50 ml of the same medium. Floating diatom cells and adhered cells onto the Petri dish surfaces were removed by this process. Incubation was carried out under a light source (27 W) at 20°C. The distance between the light and the dishes were 20 cm.

**Figure 2:**
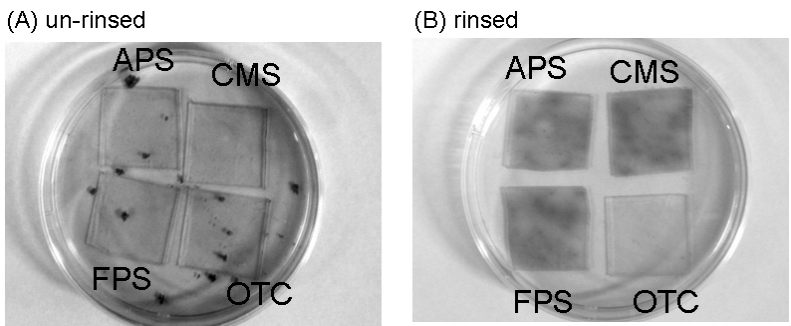
Photographs of diatoms cultured for 40 days. Glass surfaces functionalized with four types of SAMs (FPS (-CF_3_), OTC (-CH_3_), CMS (-COOH), and APS (-NH_2_)) were placed in one dish in order to unify the culture condition. (A) unrinsed. (B) rinsed after one day of incubation. The dish was 90 mm in diameter.

Figure 2 shows photographs of the above mentioned samples in one petri dish that were cultured for 40 days. Dark objects in the dish represent the grown diatom cells. In the case of unrinsed samples (Fig. 2A) that was no significant difference among the four types of functionalized glass surfaces. Many aggregates of diatom cells were found floating in the dish; these had not adhered to the glass surfaces. In general, such aggregates appeared in the control sample, in which diatoms were cultured in the usual liquid medium without glass surfaces. The data clearly showed that the floating diatom cells not adhered ones mainly grew in the case of unrinsed samples.

On the other hand, the diatom aggregates did not appear in the case of the rinsed samples (Fig. 2B). Diatom cells were successfully cultured only on the glass surfaces. Interestingly, the number of diatom cells was rather small on OTC SAMs.

From this result, we concluded that diatoms can grow on the chemically-modified glass surfaces. This is the first example of diatom cell growth on SAMs although adhesion of diatom cells was reported previously [35]. Furthermore, it is clear that rinsing the samples after cell adhesion was important to ensure that the diatom cells remained on the glass surfaces.

**Figure 3:**
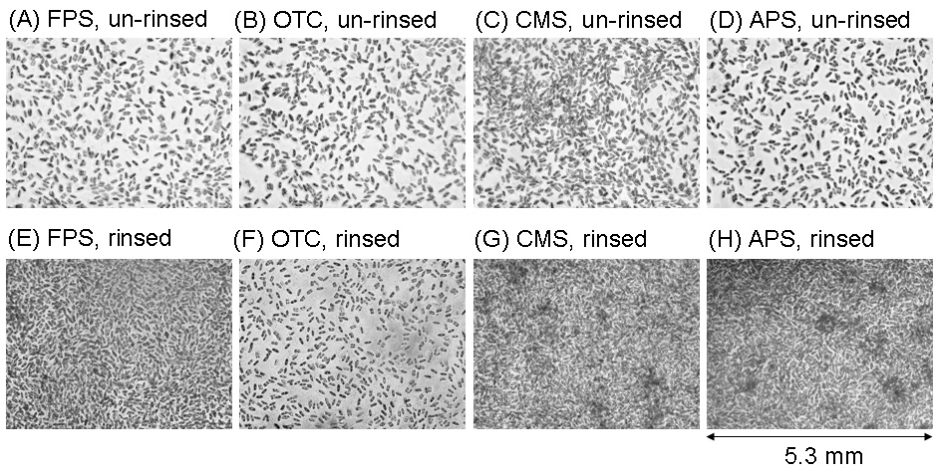
Optical microscopic images of diatom cells cultured on four types of SAM surfaces. (A), (E): FPS. (B), (F): OTC. (C), (G): CMS. (D), (H): APS. (A) to (D): unrinsed. (E) to (H): rinsed after one day of incubation. The incubation period was 40 days. Horizontal size of the images is 5.3 μm.

**Figure 4:**
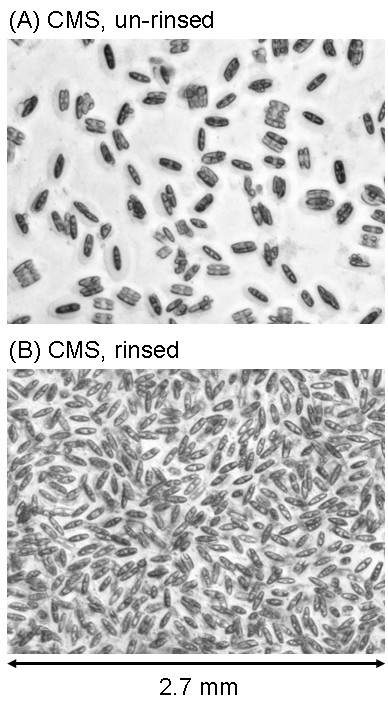
Optical microscopic images of diatom cells cultured on CMS surfaces. (A) unrinsed. (B) rinsed. The incubation period was 40 days. Horizontal size of the images is 2.7 μm.

Figure 3 shows magnified images of the diatom arrays grown on the functionalized glass surfaces. Densely packed diatom cells were observed in the case of the rinsed samples, except for OTC (Fig. 3E–3H). On the other hand, in the case of unrinsed samples, the density of the diatom cells was obviously lower than that of the rinsed samples (Fig. 3A–3D). Figure 4 shows further magnified images of the diatom cells that grew on the CMS SAMs. Although we randomly checked more than three areas for one sample, fluctuations in growth according to the observed area were negligible. The images clearly represented the effect of the rinsing treatment on the formation of a two-dimensional diatom array. Densely packed diatom arrays were also observed on the APS and FPS surfaces as well as on the CMS surface (data not shown).

If we consider the total number of diatom cells in a Petri dish, it is obvious that the number of cells in the unrinsed samples would be substantially higher than that in the rinsed samples both before and after cultivation. However, the opposite was true only in the case of the chemically modified surfaces. This phenomenon can be explained if we assume that the growth of suspended cells has priority over that of cells adherent on the chemically modified surfaces. In the case of the unrinsed samples, the adherent cells could not grow because the growth of floating cells had priority. On the other hand, in the case of the rinsed samples, the adherent cells grew well because there were no floating cells. In order to obtain detailed information about growth on the chemically modified surfaces, we observed the initial stage of cell growth by using optical microscopy in the subsequent experiments.

**Figure 5:**
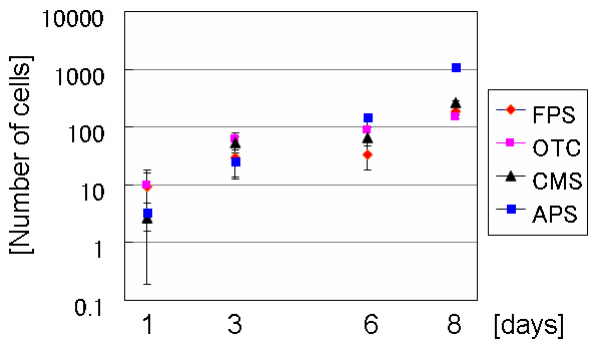
The number of cells that grew on SAM surfaces at the initial stage of incubation. The samples were rinsed with the culture medium after one day of incubation in order to remove any unattached cells. Subsequently, the samples in a single petri dish were observed after 3, 6, and 8 days of incubation.

In the case of the rinsed samples the growth rate of the diatom cells at the initial stage on SAMs was examined by counting the cell numbers. We examined the growth rate of the diatom cells at the initial stage on SAMs by counting the cell numbers in the case of the rinsed samples (Fig. 5). The number of diatom cells from one to eight days post incubation was directly counted from three randomly selected images in each sample. No significant difference was observed among the FPS, APS, CMS, and OTC surfaces on the first 6 days post incubation. Especially in the case of one day incubation, standard deviation of the data was huge. Even we verified the data using the Student's *t*-test, there was no meaningful difference among the four types of surfaces for one day incubation.

After eight days of incubation, the number of cells increased, especially on the APS-treated glass surface. After a longer incubation period, it was impossible to count the cells because the surface was too crowded. It was also difficult to count the cells in the unrinsed samples because there were many aggregates.

The data clearly demonstrated that the number of cells on the OTC-treated surface was not less than that of on the CMS and FPS surfaces after eight days incubation. However, after 40 days incubation, the number of cells was considerably lower than that on the FPS, CMS, and APS surfaces.

The number of cells on APS-treated surface was not much different from others within six days of incubation. However, after eight days, concentration of cells on the APS surface was higher than others. We confirmed that the difference was meaningful by the *t*-test. As one of the differences between APS and other compounds, only APS has positive charge in the culture medium. As one possibility, we speculate that charge of the surfaces give some effect on cell growth. The phenomena on OTC and APS-treated surfaces were interesting although further experiments are necessary to understand the mechanism.

**Figure 6:**
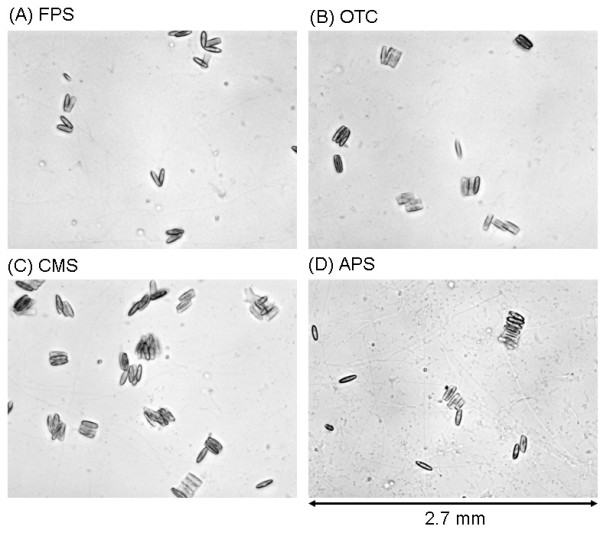
Optical microscopic images of diatom cells on SAM surfaces incubated for 3 days. (A) FPS. (B) OTC. (C) CMS. (D) APS. The sample was rinsed after one day of incubation. Horizontal size of the images is 2.7 μm.

Figure 6 shows specific structures that were observed at three days of incubation in the case of the rinsed samples. At one day of incubation, most of the cells were individually isolated (data not shown). However, after three days, clusters of diatom cells were observed throughout each of the SAM surfaces. Some of the clusters were three dimensional, but most were two-dimensional clusters.

The data can allow several speculations. Firstly, diatom cells were probably trapped on the SAM surfaces; thus, a higher number of cells formed clusters on the surfaces. Secondly, some cells may have retained their motility because the diatom cells ultimately formed two-dimensional clusters and not three-dimensional ones. One possible explanation is as follows: if a diatom cell grew on another diatom cell and not on a SAM surface, the grown cell might have motility on the other diatom cell surface. On the other hand, it was not possible for the diatom cell at the bottom to move because it was attached to the SAM surface. The upper cell can move on the diatom cell surface; however, it is trapped when it comes in contact with the SAM surface. Thirdly, two-dimensional growth was not common in the case of the unrinsed samples at this stage (data not shown). As we discussed before, results in Figure 2 suggested that floating cells probably had a higher priority of growth in contract to adhered cells. The result in Figure 6 supports the speculation. Even in the unrinsed samples, some of the diatom cells must be adhered onto the chemically modified surfaces although two-dimensional growth was not common. It suggests that growth of the adhered cells were not fast when floating cells are coexistent.

**Figure 7:**
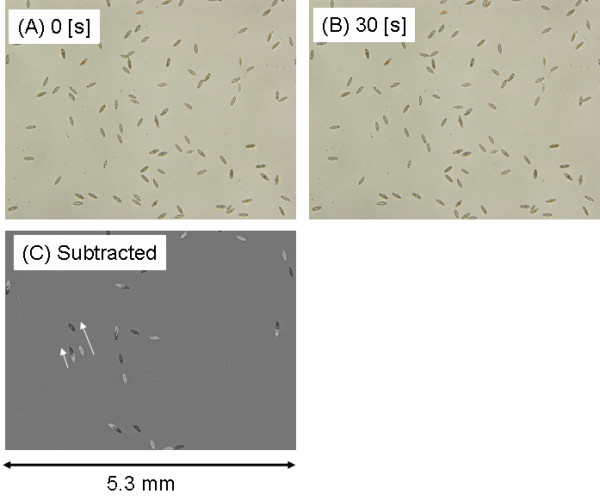
An example of subtracting two images that were captured by time-lapse optical microscopy. (A) Initial image. (B) The same area with (A) captured after 30 s. (C) subtracted image of (A) and (B). Three days incubation on the CMS SAM without rinsing. Horizontal size of the images is 5.3 μm.

The motility of diatom cells on the SAM surfaces was investigated by time-lapse observations in order to verify the interaction between the diatom cells and the SAM surfaces. A total of 50 optical microscopy images were continuously captured every 30 s for each area. Subsequently, two adjacent images were subtracted as explained in Figure 7. A typical example of the subtracted images using an unrinsed sample with the CMS-treated surface was shown in Figure 7C. If a diatom cell did not move for 30 s, the cell was deleted by the subtraction. For a cell that moved during this period, a bright and a dark shadows represented the initial and final position of the cell. The distance moved was measured as the length of the two shadows. In this example, there were almost 95 cells in Figure 7A and 7B. Among these cells, almost 15 cells were moved as shown in Figure 7C.

**Figure 8:**
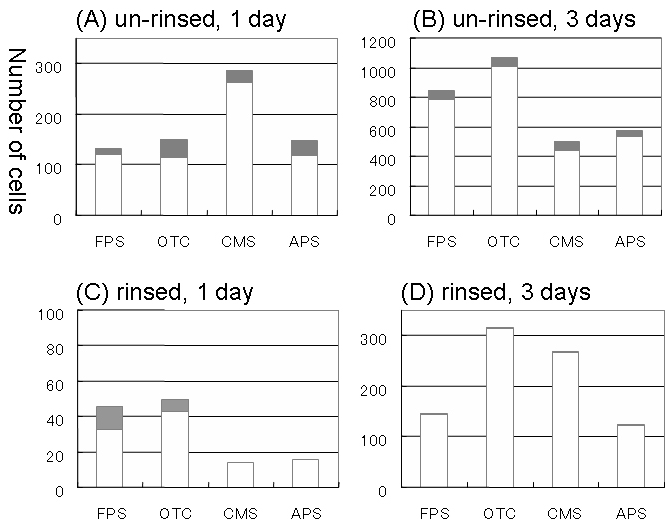
Histogram of the moved and unmoved diatom cells on SAM surfaces. (A) One day of incubation, unrinsed. (B) Three days of incubation, unrinsed. (C) One day of incubation, rinsed. (D) Three days of incubation, rinsed. White and gray bars show unmoved and moved cells, respectively.

Figure 8A and 8B show the number of moved and unmoved cells counted from the optical microscopic images of the unrinsed samples. Cells that moved were observed on all surfaces after both one day and three days, respectively, of incubation although more than 90% of the cells had not moved. Since only a few cells were moved in the rinsed samples, quantitative discussion of this data is not suitable. The graph showed the rough ratio of moved cells to unmoved cells. Qualitatively, it was clear that moved cells were no longer observed in the rinsed samples after three days of incubation. However, cells grew well on the surfaces as shown in Figures 2, 3, and 4. This indicates that the diatom cells attached on the surfaces were active even those were not moved.

**Figure 9:**
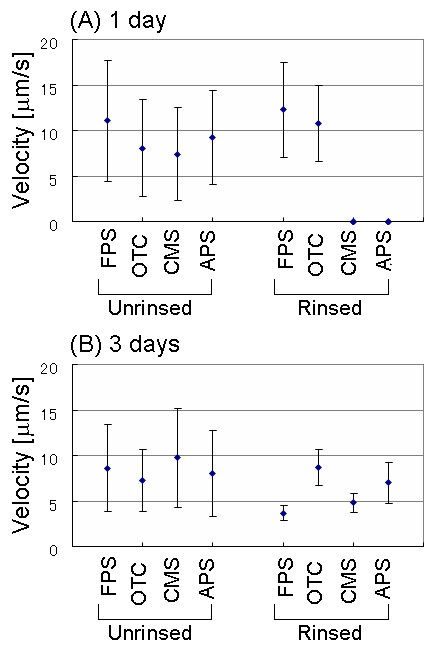
The velocity of diatom cells moving on SAM surfaces. (A) One day of incubation. (B) Three days of incubation.

The velocities of the moving cells are plotted in Figure 9. Data was obtained from three independent images for each surface, and the average value was then calculated. In the case of the unrinsed samples, the velocities of diatom cells on the FPS, OTC, CMS, and APS-treated surfaces were 84.9 ± 51.0, 62.0 ± 40.7, 56.9 ± 39.4, and 70.8 ± 39.5 μm/s, respectively, after one day of incubation. After three days of incubation, the velocities were 66.0 ± 36.7, 55.7 ± 26.2, 74.7 ± 41.5, and 61.6 ± 36.6 μm/s, respectively. The velocities at one and three days of incubation were verified using the *t*-test. Although the velocities fluctuated greatly, the decrease in velocity after three days of incubation was meaningful for the FPS and OTC samples at a 5% level of significance.

On the other hand, in the case of the rinsed samples, a few cells moved on FPS and OTC when the samples were observed just after rinsing (one day of incubation). The velocity was 94.2 ± 39.8 and 82.8 ± 32.3 μm/s on FPS and OTC surfaces, respectively. Since the total number of diatom cells after rinsing was almost less than ten per area, the values obtained must be considered just as examples, as there were not enough data points to create a meaningful average. No moving cells were observed on APS and CMS SAMs.

When the same samples were observed after three days of incubation, moving cells were detected on all the surfaces, although the number of moving cells was very few. The velocity of the moving cells was 28.2 ± 6.3, 66.7 ± 14.9, 36.7 ± 7.9, and 53.6 ± 17.3 μm/s on FPS, OTC, CMS, and APS surfaces, respectively. On the other hand, when the same samples were observed after six days of incubation, no moving cells were observed at all.

Our data revealed that the velocity of the diatom cells in the unrinsed samples was higher than that in the rinsed samples after three days of incubation. The higher velocity of the cells in the unrinsed samples was easy to understand because this sample had many unattached cells. On the other hand, it was interesting that some of the cells in the rinsed samples could move after three days of incubation, although very few cells moved at the initial stage. Newly grown cells have some mobility even on SAM surfaces. However, the velocity of the cells in the rinsed samples was much lower than that of the cells in the unrinsed samples. This suggests that the SAM surfaces have the potential to regulate diatom cell motilities, although the fluctuation was rather large in our experiments. There was no significant difference between the four types of SAMs examined in this study.

The chemically modified glass surfaces were examined by atomic force microscopy (AFM), static water contact angle measurement, X-ray photoelectron spectroscopy (XPS), and Fourier transform infrared spectrometer (FT-IR). Static water contact angles (θ_w_) of FPS, OTC, CMS, and APS were 43.7 ± 2.6, 58.4 ± 4.5, 23.2 ± 4.3, and 62.2 ± 1.1 degrees, respectively. The results suggested that the APS and OTC surfaces were rather hydrophobic. The FPS surface exhibited an intermediate value, although it was much higher than the CMS-treated surface. If the surface was completely covered with -CF_3 _groups, a much higher value can be expected because of the hydrophobic property of the -CF_3 _groups. This suggests that in our experiments, the surface was not entirely covered with -CF_3 _SAM because we selected relatively mild condition for silanization (dipping for 30 min in 0.1% solution). Studying the effect of silanization conditions on cell growth is probably an interesting subject for the future research.

By XPS measurements, N and F were detected from the APS and FPS samples, respectively, although the signals were weak. In the case of the OTC and CMS samples, a specific signal could not be detected although C was detected. In FT-IR measurements, the CMS sample showed a specific signal of the -COOH group at approximately 1700 cm^-1^. The APS sample was also demonstrated a signal of the -NH_2 _group at 1560-1640 cm1^-1^. In both the XPS and FT-IR measurements, the signals obtained were not adequately intense for quantitative discussion.

**Figure 10:**
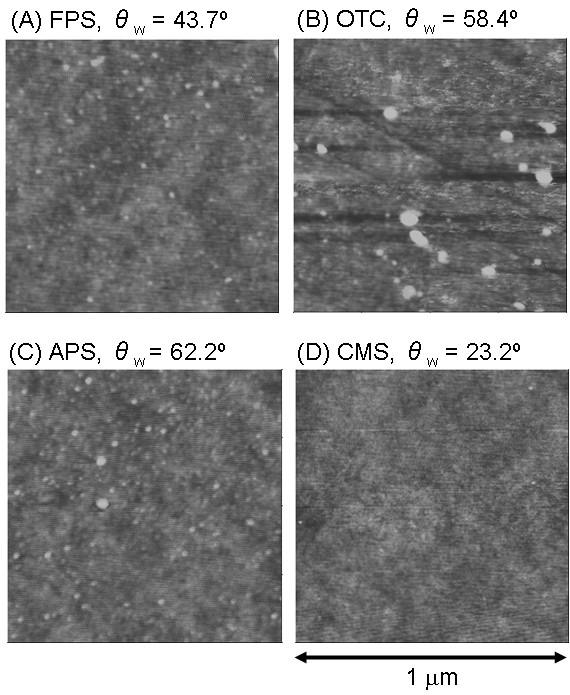
AFM images of chemically modified glass surfaces. (A) FPS, (B) OTC, (C) APS, and (D) CMS. Scan size was 1 μm. Values on each image showed static water contact angle (θ_w_).

Figure 10 shows typical AFM images of the chemically modified surfaces. The CMS surface exhibited the flattest features. Thus, CMS treatment yielded a flat and hydrophilic surface. Under neutral pH conditions, the surface should have a negative charge due to the -COOH groups. The AFM images of APS and FPS samples showed that they had almost flat surfaces with small aggregates. In the case of the APS sample, a flat and hydrophobic surface was observed. The surface should have a positive charge due to the -NH_2 _group. FPS treatment yielded flat and slightly hydrophobic surface without any charge. Finally, the AFM images of OTC-treated surface showed that it had the roughest surface. Aggregates that were several tens of nm in diameter were present ubiquitously.

## Conclusion

We demonstrated the fabrication of a two-dimensional array of densely packed living diatom cells by using four types of chemically modified surfaces. When excess diatom cells were removed by rinsing before starting the long term incubation, two-dimensional arrays of densely packed cells were realized on the FPS, APS and CMS SAM surfaces. Furthermore, the analysis of the adhesion of diatom cells on the SAM surfaces and their motility on these surfaces provided fundamental information for preparing better biodevices with living diatom cells.

## Materials and methods

A marine diatom, *Navicula sp*., was cultured with Daigo's IMK culture medium (Nihon Pharmaceutical Co. Ltd., Osaka, Japan) in sea water. The sea water was taken at Arasaki coast (Kanagawa, Japan), and kept longer than three months prior to use. Na_2_SiO_3 _(1 mM; Wako Pure Chemical Industries, Ltd., Osaka, Japan) was added to the culture medium as a Si source.

7-Octenyltrichlorosilane (OTC), 2-(carboxymethylthio)ethyl 3-trimethylsilane (CMS), and 3,3,3-(trifluoropropyl)trimethoxysilane (FPS) were purchased from Gelest Inc (PA, USA). 3-Aminopropyltriethoxysilane (APS) was bought from Shin-Etsu Chemical Co., Ltd. (Tokyo, Japan).

For preparing SAMs on glass surfaces, glass substrates were immersed in 0.1% of OTC, CMS, FPS, or an APS ethanol solution for 30 min at room temperature [20,21]. The glass was washed with ethanol prior to functionalization. The substrates were subsequently rinsed with ethanol three times, and then baked at 90°C for 1 h. Finally, the substrates were rinsed with ethanol again.

The chemically modified surfaces were characterized by AFM (NanoScopeIIIa Digital Instruments Inc., Santa Barbara CA), static water contact angle meter (CA-X, Kyowa Interface Science Co. Ltd., Saitama, Japan), XPS (SSX-100, Surface Science Instruments, Mountain View, CA), and FT-IR (Spectrum One, PerkinElmer, Waltham, MA).

All the functionalized glass substrates (OTC, CMS, FPS, and APS) were put in one polystyrene dish (90 mm in diameter). Fifty ml of the culture medium including Si was injected into the dish. The precultured diatom suspension was then added to the dish.

The samples were incubated at 20°C under a fluorescent light (27 W, FPL27EX-N, Hitachi, Tokyo, Japan). Distance between samples and the light was 20 cm. Some of the samples were rinsed with the culture medium in order to remove unattached cells after 24 h incubation. When the incubation was continued for longer than two weeks, pure water was added to the dishes to avoid drying.

Samples were directly observed with an optical microscope (IX71, Olympus, Japan) at 20°C on after 1, 3, 6 and 8 days of incubation. After 40 days of incubation, the samples were again observed by optical microscopy for the final characterization. After the number and velocity of the cells were measured from the microscopic images, three randomly selected images were employed. ImageJ ver.1.36b (National Institutes of Health (NIH), Bethesda, MD) was used for analyzing motility of diatom cells with the microscopic images.

## Competing interests

The author(s) declare that they have no competing interests.

## Authors' contributions

KU and TY did most of experiments and data analysis in the laboratory. YM and KK did XPS, FT-IR, and water contact angle measurements. KU, KR, and SM coordinated experiments, and provided important advice for the experiments. SM gathered the diatoms at Chiba prefecture in Japan.
